# Recurrent stress urinary incontinence surgery in the United Kingdom: an analysis of the British Society of Urogynaecology database (2007–2015)

**DOI:** 10.1007/s00192-020-04420-3

**Published:** 2020-07-23

**Authors:** Dina El-Hamamsy, Douglas G. Tincello

**Affiliations:** 1 University Hospitals of Leicester NHS Trust, Leicester, England; 2 Department of Health Sciences, College of Life Sciences, University of Leicester, University Road, Leicester, LE1 7RH UK

**Keywords:** Recurrent incontinence, Repeat surgery, Stress incontinence, Urinary incontinence

## Abstract

**Introduction and hypothesis:**

There is a lack of robust evidence guiding treatment options for recurrent stress urinary incontinence (SUI) and limited comparative outcome data. The aim of this study was to examine the pattern of surgery for recurrent SUI performed by gynaecologists in the UK and compare subjective success rates.

**Methods:**

Retrospective review of the British Society of Urogynaecologists database for patients having repeat incontinence procedures (2007–2015) including the number of each procedure and outcome recorded by the International Consultation on Incontinence Urinary Incontinence Short Form (ICIQ-UI-SF) questionnaire. Procedures were compared by year and outcomes by operation. Categorical comparisons were performed using Chi-squared test and numerical comparisons using appropriate non-parametric tests.

**Results:**

A total of 2,938 records were obtained (269 were excluded) and 2,164 women (88.8%) had undergone one previous procedure, most commonly retropubic midurethral sling (MUS; 28.6%). Pelvic floor exercises were offered to 76.2% women. Urodynamic investigation was carried out in 96.2% women: 76.5% had urodynamic stress incontinence. Repeat MUS was the most common procedure (77.3%), followed by bladder neck injections (BNI; 10.2%). Follow-up details were available for 66.1%. Outcome data were poorly reported. Median ICIQ-UI-SF score fell from 16 (0–21) to 0 (0–21) (*p* < 0.001), 81.6% felt “much better” or “very much better” on Patient Global Impression of Improvement (PGI-I), and 89.3% “cured” or “improved”. MUS, colposuspension and fascial sling showed the best results with regard to the PGI-I score and “change in SUI” (*p* < 0.001).

**Conclusion:**

MUS and BNI were the most common repeat continence procedures. Follow-up data suggest that MUS, colposuspension and fascial sling are most effective.

## Introduction

Urinary incontinence occurs in up to 42% of women, with Stress Urinary incontinence (SUI) accounting for about half of the cases, with a significant proportion being “socially disabling” [1, 2].

For women in whom conservative measures are ineffective, a variety of surgical treatments are effective in about 60–90% of cases [3]. Thus, 10–40% of women experience failure or recurrence and many opt for repeat surgery. Population-based studies have shown an incidence of repeat surgery for SUI of 3.9 to 14.5% [4–7]. The incidence of repeat surgery differed by type of procedure, from 61.2% up to 9 years following bladder neck injections (BNIs), through 22.2% after needle suspension, to 13.0% after a fascial sling, and 10.8% after Burch colposuspension [6].

Currently, treatment of recurrent SUI is varied and largely based on surgeon’s preference and experience, which may contradict women’s expectations and preferences [8]. There is a lack of robust evidence guiding treatment options for recurrent SUI. A recent Cochrane review has failed to identify evidence to support or refute any management strategy for recurrent SUI after failed midurethral slings [9], and other recent systematic reviews have extracted data on subsets of women with recurrent SUI [10, 11]. There are no published randomized trials of sufficient power comparing outcomes in women with recurrent SUI. Evidence from non-randomized studies suggests a success rate of about 73–79% after midurethral tapes for recurrent surgery, and data from the systematic reviews show a wide range of cure between 39 and 100% [10, 11].

We aimed to investigate types of surgery for recurrent SUI in the UK from 2007 to 2015, as recorded on the British Society of Urogynaecologists (BSUG) database and compare their patient-reported outcomes.

## Materials and methods

This study was based on data routinely collected by the BSUG; thus, ethical approval was not required. BSUG is a section of the Royal College of Obstetricians and Gynaecologists (RCOG) that is specifically dedicated to urogynaecology, and was founded in 2001. Although highly encouraged, data input to the BSUG database is currently voluntary in the UK and is only available to subscribing members. As of October 2017, there were 486 members. Surgeons involved included gynaecologists with a special interest in urogynaecology, subspecialist urogynaecologists, and their trainees. Permission to access and use the data was obtained from the BSUG database committee. Data collected contained no patient-identifiable information. However, patients whose data were collected routinely signed a BSUG consent form before their data were collected. No specific funding was obtained for this study.

Patients recorded as having recurrent stress urinary incontinence (SUI) were extracted from the database. The details of how SUI was diagnosed are not included in the database; they was entered by the host surgeon. In the UK, SUI was usually diagnosed on the basis of urodynamic assessment, as per current NICE guidance, but we were unable to check the diagnosis for individual patients. Data were then cleaned removing duplicate cases, cases that did not have continence procedures and cases that were not recurrent. The total number of procedures and the relative frequency of each procedure were compared by year and the outcomes of each continence procedure type were compared by patient-reported clinical outcomes. These included Patient Global impression of Improvement (PGI-I) [12], change in SUI, as reported on a four-point Likert scale on the database (worse; no change; improved; cured), and the validated International Consultation on Incontinence modular Questionnaire Urinary Incontinence Short Form (ICIQ-UI-SF) [13]. We were unable to confirm which specific bladder neck injection agent was used. Data were presented using descriptive statistics. They were compared using Chi-squared test for categorical data, and Mann–Whitney* U* and Wilcoxon Signed Rank tests for continuous data.

## Results

Records were obtained for 2,938 cases between 2007 and 2015 (the year the database was opened, up to when the data were extracted for this analysis), of which 269 records were excluded during the data cleaning process. Data were analysed for 2,669 cases, although in 231 cases details of previous surgery were missing. The median age of the patients at the time of surgery was 59 years (range: 20–88) and the median BMI was 28.4 kg/m^2^ (range: 17.8–60.6; Table 1).

**Table 1:** Basic demographic data

Age, median (range)	BMI, median (range)	Number of previous procedures:* n* (%)	First procedure,* n* (%)
59 years (20–88)	28.4 (17.8–60.6)	One: 2,164 (88.8)	Retropubic midurethral sling: 698 (28.6)
		Two: 207 (8.5)	Transobturator midurethral sling: 425 (17.4)
		Three: 53 (2.2)	Colposuspension: 597 (24.5)
		Four or more: 14 (0.6)	Bladder neck injection: 349 (14.3)

Data on previous procedures were available in 2,438 patients. The number of previous continence procedures was 1 in 2,164 women (88.8%), 2 in 207 (8.5%), 3 in 53 (2.2%) and ≥ 4 in 14 (0.6%) patients (Table 1). Previous procedures were most commonly retropubic midurethral sling (MUS) in 698 women (28.6%), followed by colposuspension in 597 (24.5%), transobturator MUS in 425 (17.4%), and bladder neck injections in 349 (14.3%) patients (Table 1). Fascial slings had been performed in 89 (3.7%) and anterior colporrhaphy with bladder neck buttress suture in 87 (3.6%) cases, while 7 (0.3%) cases were grouped as “other procedures”, e.g. laparoscopic urethropexy or artificial urinary sphincter.

Pelvic floor muscle training was offered to 1,725 out of 2,264 patients (76.2%) where that data field was complete, of which 135 patients (6.0%) declined it. Urodynamic investigation was performed in 2,280 out of 2,370 cases (96.2%) where that data field was complete, and showed urodynamic stress incontinence (USI) in 1,802 (76.5%), mixed incontinence in 439 (18.6%), detrusor overactivity (DO) in 16 (0.7%) and the results were normal in 80 cases (2.2%).

**Fig. 1:**
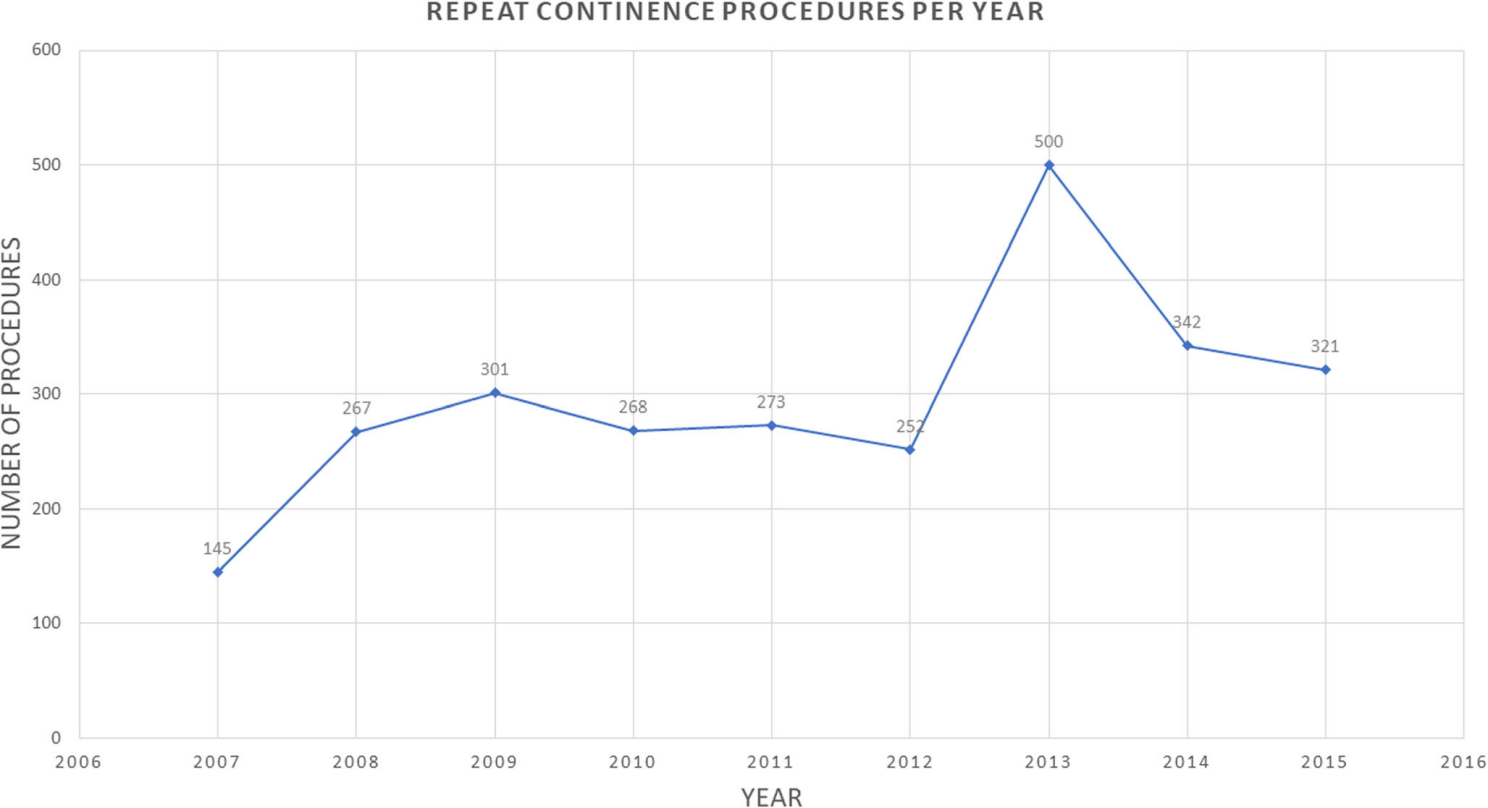
Numbers of recurrent SUI procedures recorded on the British Society of Urogynaecologists database (2007–2015)

The median (range) number of repeat continence procedures per year was 273 (145–500) procedures. There was a non-linear increase across the years, peaking at 500 in 2013 (Fig. 1). That year, the UK government requested a national audit of continence procedures from the BSUG, and reflects higher database uploads for that year. Overall, MUS (including both retropubic and transobturator approaches) was the most commonly performed repeat procedure (2,062; 77.5%), followed by BNI (272; 10.2%), colposuspension (152; 5.7%), fascial sling (89; 3.3%), anterior repair and bladder neck buttress suture (89; 3.3%), and other procedures (7, 0.3%).

**Fig. 2:**
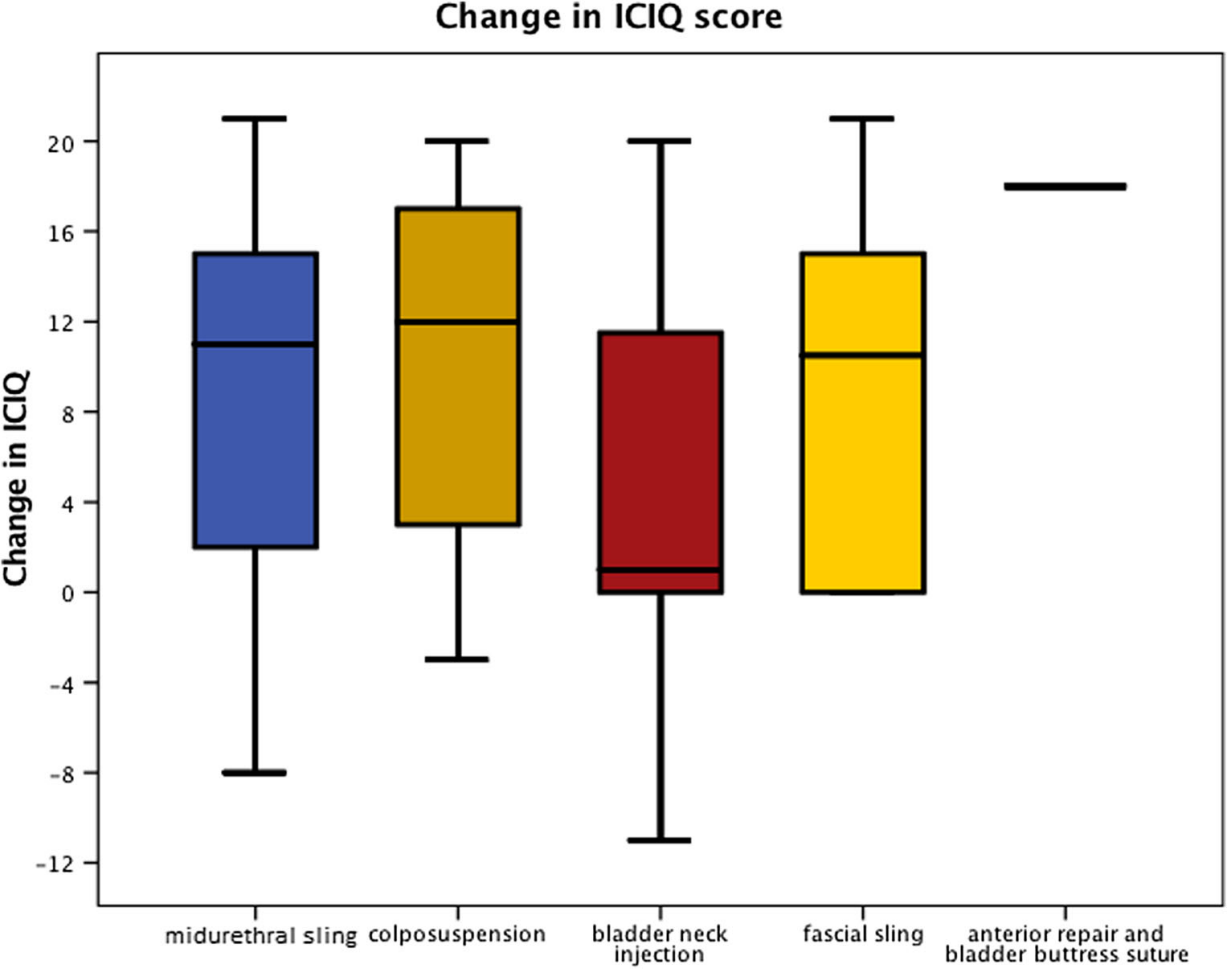
Change in International Consultation on Incontinence modular Questionnaire Urinary Incontinence Short Form (ICIQ-UI-SF) by procedure

Follow-up details were available for 1,763 patients (66.1%). The method of follow-up was via the outpatient clinic in 1,573 (89.2%), postal questionnaire in 100 (5.7%) and the telephone in 90 cases (5.1%). The follow-up interval was documented in 1,734 (65.0%) cases and was 6 weeks in 649 (37.4%), 3 months in 667 (38.5%), 6 months in 354 (20.4%) and 12 months in 64 cases (3.7%). The PGI-I was reported in 1,616 cases (60.5%). It was “very much better” in 988 (61.1%), “much better” in 331 (20.5%), “a little better” in 124 (7.7%), there was “no change” in 128 (7.9%), it was “a little worse” in 19 (1.2%), “much worse” in 19 (1.2%) and “very much worse” in 7 cases (0.4%). Subjective change of SUI was reported in 1,497 (56.1%). Patients reported being “cured” in 992 (66.3%), “improved” in 344 (22.9%), “no change” in 117 (7.8%), “worse” in 21 (1.4%) and that SUI was “never present” in 23 cases (1.5%; Table 2). The preoperative ICIQ-UI-SF was reported for 882 (33.0%) and the postoperative one in 621 (23.3%) women. The median score fell from 16 (0–21) preoperatively to 0 (0–21) postoperatively (*p* < 0.0001). All measures of improvement (PGI-I, change of SUI, change in ICIQ-UI-SF) differed according to the type of procedure (Fig. 2; Table 3 ).

**Table 2:** Outcome data by global impression and change in stress incontinence

Global Impression	Number (%) [1,616]	Dichotomised %	Change in stress incontinence	Number (%) [1,499]	Dichotomised %
Very much better	988 (61.1)	81.6	Cured	993 (66.3)	89.1
Much better	331 (20.5)		Improved	344 (22.9)	
A little better	124 (7.7)	18.4	No change	117 (7.8)	9.2
No change	128 (7.9)		Worse	21 (1.4)	
A little worse	19 (1.2)				
Much worse	19 (1.2)				
Very much worse	7 (0.4)				

Numbers in square brackets indicate the number with completed data

**Table 3:** Outcome data by procedure

Procedure	PGI-I: “better/very much better”, number (%)	Change in stress: “cured/improved”, number (%)	Change in ICIQ-UI-SF median (range)
MUS	1,111 (85.1)	1,124 (91.7)	11 (−8 to 21)
Colposuspension	80 (77.7)	88 (90.7)	12 (−3 to 20)
Fascial sling	41 (89.1)	31 (93.9)	10.5 (0 to 21)
BNI	73 (51.0)	84 (65.1)	1 (−11 to 20)
Anterior repair and bladder neck buttress suture	11 (73.3)	9 (69.3)	18^a^
*p*	0.0001	0.0001	0.007

*MUS* midurethral sling,* BNI* bladder neck injection, *ICIQ-UI-SF* International Consultation on Incontinence modular Questionnaire Urinary Incontinence Short Form

^a^Only one case reported

## Discussion

There was an overall increase in the provision of repeat continence procedures in the UK across the years, with numbers more than doubled between 2007 and 2015. This increase could be explained either by a true rise in the rate or improved documentation on the BSUG database due to increased awareness, patient pressure and government initiatives.

Our findings are consistent with those of the international literature. In the UK (this study), repeat procedures were 77.3% MUS, 10.2% BNI and 5.7% colposuspensions. Jonsson Funk et al. reported corresponding proportions of 70.5%, 20.1% and 6.5% respectively in the USA [6].

Data from the USA, Taiwan and Canada show that MUS are the most commonly performed secondary continence procedures, with percentages ranging between 50 and 80% [6, 7, 14]. Wu et al. also reported that 63.5% of patients in Taiwan who had MUS as a primary procedure chose to have it as a secondary procedure [7]. This may be related to surgeons’ training and experience [8, 15].

One of the limitations of this analysis is the high proportion of MUS as the repeat procedure (77.3% of our sample). While we can be reasonably confident of the data for these procedures, we should apply caution in the comparison between these and the other procedures, because they represent 10% or less of the whole cohort. Having said this, the outcomes do broadly reflect the relative efficacy of these procedures when performed as primary surgery.

This was a UK-wide study, based on a national database, whose validity has been demonstrated (53) and included a large population, with the benefit that the success of repeat continence procedures was recorded with validated patient-reported outcomes. These have been validated against other tools measuring subjective patient outcomes [16]. However, the completeness of the BSUG database reporting was overall poor. Follow-up data were documented in 66.1%, PGI-I in 60.5%, change of SUI in 56.1% and postoperative ICIQ-SF in 23.3%. Further, we acknowledge the relatively short-term follow up from this dataset, with two-thirds of patients having only short-term follow-up (6 weeks or 3 months), and only 24% having data beyond 6 months. This incomplete reporting needs to be taken into account when interpreting the results, and also should be highlighted as an area where greater diligence is needed to ensure collection and recording of outcomes during follow-up of patients in routine practice. Additionally, at present, the registering of patients on the database is voluntary; thus, there is a degree of responder bias, given that it is inevitable that not every patient has been registered. The patient outcomes in the database are reported by the patient (usually via face-to-face or telephone consultation); thus, there is also likely to be a degree of responder bias from them. These facts contribute a further degree of uncertainty to the data. A mandatory, nationwide registry is currently under discussion in the UK that should address such problems in the future [17].

Another factor to bear in mind when interpreting these data is that the BSUG database did not comprehensively record whether patients had SUI alone, or mixed UI. Also, there was no data field to record if the underlying aetiology was urethral hypermobility or intrinsic sphincter deficiency. The lack of aetiology does limit interpretation somewhat, although the fact that the majority of cases involved MUS, which is known to be effective regardless of aetiology, mitigates against this [18, 19].

Success varied by the type of procedure performed, with MUS, colposuspension and fascial slings having comparable results and overall were more effective for recurrent SUI than BNI or anterior repair and bladder neck buttress suture (Fig. 2; Table 3), as shown by all three outcomes (PGI-I, subjective cure/improvement and ICIQ-UI-SF scores). This is important to aid counselling in the absence of robust randomised controlled trial (RCT) data.

Studies have shown a wide range of success rates of about 40–100% for MUS, colposuspension and fascial slings [10]. However, most studies reporting specifically on MUS as a repeat continence procedure were small, with short-term follow-up, and used a variety of definitions of success. Parden et al. reported that although the success rates of repeat MUS are lower than those of primary MUS procedures, they result in greater improvement of QoL, and explained that with a worse baseline SUI in those patients [20].

In a recent study comparing outcomes of different repeat continence procedures, Cerniauskiene et al. have also reported that colposuspension and MUS have comparable and good success rates [21].

Studies have reported success rates for colposuspension of 55–93%, with subjective measures generally reporting higher success rates than objective measures [22–25]. A 2015 systematic review showing a pooled success rate of 76% (95% CI ± 5.04) [26].

Lee et al. reported a success rate of secondary fascial slings of 65.7% using patient-reported outcomes and no reoperation as a definition of success [27]. Amaye-Obu and Drutz had reported objective and subjective cure rates for abdomino-vaginal polypropylene sling of 78% and 89%, “modified” urethral sling of 70% and 96%, and colposuspension of 73% and 88% respectively. More importantly, they observed declining success with a higher number of previous continence procedures [28].

Gaddi et al. reported a failure rate of BNI as a repeat continence procedure of 38.8%, i.e., success of 61.2%, where the definition of failure included both subjective and objective measures [29]. Futyma et al. reported a 24-month 32.7% objective success rate using non-absorbable material, whereas Zivanovic et al. reported 25.4% and 58.2% cure and improvement rates at 12 months using polyacrylamide hydrogel [30, 31]. Isom-Batz and Zimmern reported 93% initial subjective cure/improvement rates with collagen injection; however, previous urethral surgery included non-incontinence procedures and a further 6 out of 31 patients (19.4%) had progressive failure and dissatisfaction over time [32].

Despite the current media and public concerns about synthetic mesh implants, MUS remained the most commonly performed procedure for recurrent SUI in the UK (until the temporary suspension enforced in 2018), with a median yearly proportion of 76.8% (range: 62.3 to 85.4%).

Overall, repeat MUS, colposuspension and fascial sling procedures appear to be the best secondary procedures, based on this large cohort. Admittedly, there are few RCT data, but given the current concerns regarding mesh, these data are important to help clinicians to advise and counsel patients while RCT data from ongoing studies are awaited.
